# Epigenome-wide DNA methylation in obsessive-compulsive disorder

**DOI:** 10.1038/s41398-022-01996-w

**Published:** 2022-06-01

**Authors:** Miriam A. Schiele, Jan Lipovsek, Pascal Schlosser, Michael Soutschek, Gerhard Schratt, Michael Zaudig, Götz Berberich, Anna Köttgen, Katharina Domschke

**Affiliations:** 1 Department of Psychiatry and Psychotherapy, Medical Center—University of Freiburg, Faculty of Medicine, University of Freiburg, Hauptstraße 5, 79104 Freiburg, Germany; 2 Institute of Genetic Epidemiology, Faculty of Medicine and Medical Center—University of Freiburg, Hugstetter Straße 49, 79106 Freiburg, Germany; 3 Department of Epidemiology, Johns Hopkins University Bloomberg School of Public Health, Baltimore, MD USA; 4 ETH Zurich–D-HEST, Institute for Neuroscience, Systems Neuroscience, Building Y17 L48, Winterthurerstraße 190, 8057 Zurich, Switzerland; 5 Psychosomatic Hospital Windach, Schützenstraße 100, 86949 Windach, Germany; 6 Center for Basics in NeuroModulation, Faculty of Medicine, University of Freiburg, Breisacher Straße 64, 79106 Freiburg, Germany

**Keywords:** Biomarkers, Epigenetics in the nervous system

## Abstract

In adult patients with obsessive-compulsive disorder (OCD), altered DNA methylation has been discerned in several candidate genes, while DNA methylation on an epigenome-wide level has been investigated in only one Chinese study so far. Here, an epigenome-wide association study (EWAS) was performed in a sample of 76 OCD patients of European ancestry (37 women, age ± SD: 33.51 ± 10.92 years) and 76 sex- and age-matched healthy controls for the first time using the Illumina MethylationEPIC BeadChip. After quality control, nine epigenome-wide significant quantitative trait methylation sites (QTMs) and 21 suggestive hits were discerned in the final sample of 68 patients and 68 controls. The top hit (cg24159721) and four other significant QTMs (cg11894324, cg01070250, cg11330075, cg15174812) map to the region of the microRNA 12136 gene (*MIR12136*). Two additional significant CpG sites (cg05740793, cg20450977) are located in the flanking region of the MT-RNR2 (humanin) like 8 gene (*MT-RNRL8*), while two further QTMs (cg16267121, cg15890734) map to the regions of the MT-RNR2 (humanin) like 3 (*MT-RNRL3*) and MT-RNR2 (humanin) like 2 (*MT-RNRL2*) genes. Provided replication of the present findings in larger samples, the identified QTMs might provide more biological insight into the pathogenesis of OCD and thereby could in the future serve as peripheral epigenetic markers of OCD risk with the potential to inform targeted preventive and therapeutic efforts.

## Introduction

Obsessive-compulsive disorder (OCD) is a highly burdensome mental disorder with a lifetime prevalence of 1–3% and is associated with poor quality of life and severe functional impairment in various domains of life [1]. The etiology of OCD is considered multifactorial, comprising complex interactions between genetic factors with a heritability of 27–65% on the one hand and environmental influences on the other [2].

Epigenetic alterations seem to be particularly relevant in OCD given that while the first published genome-wide association study (GWAS) in OCD yielded no genome-wide significant results, an enrichment of methylation quantitative trait loci (meQTLs) was observed, i.e., an enrichment of genetic variation associated with changes in DNA methylation [3]. Thus far, however, investigations into the peripheral DNA methylation signatures of OCD are scarce, and efforts have mostly focused on candidate-gene approaches. For instance, differential DNA methylation patterns between OCD patients and healthy control probands have been described for the oxytocin receptor (*OXTR*) gene [4–7], the serotonin transporter (*SLC6A4*) gene [8], the monoamine oxidase A (*MAOA*) gene [9], the brain-derived neurotrophic factor (*BDNF*) gene [10], and, on a nominally significant level, the gamma-aminobutyric acid B receptor 1 (*GABBR1*), estrogen receptor 1 (*ESR1*), myelin oligodendrocyte glycoprotein (*MOG*) genes and again the brain-derived neurotrophic factor (*BDNF*) gene [11].

Applying a hypothesis-generating approach, an epigenome-wide association study (EWAS) in 59 pediatric patients (aged 4–18 years) with OCD using the Illumina Infinium HumanMethylation450 BeadChip on saliva samples identified differential methylation at cytosine-phosphate-guanine (CpG) sites in the *C13orf39*, *C17orf54*, *DNAJC15*, *LLGL2*, *POLS*, *MAD1L1*, *MGC87042*, *PTPRN2*, and *SGK2* genes to be associated with both OCD and attention-deficit hyperactivity disorder in a subset of cases (*N* = 43) with more severe symptoms as defined by clinical cutoff scores [12]. The only EWAS available so far in an adult population using the Illumina Infinium Human Methylation450 BeadChip on blood samples discerned differential methylation in 2190 genes at an adjusted *p* < 0.05 comprising, e.g., the *BCYRN1*, *BCOR*, *FGF13*, *HLA-DRB1*, and *ARX* genes in 65 Chinese Han patients with OCD compared to 96 healthy controls [13].

In the present study, epigenome-wide DNA methylation profiles in blood were investigated for the first time in a sample of adult OCD patients of European ancestry and corresponding healthy controls using the Illumina Infinium MethylationEPIC BeadChip array covering an additional ~350,000 CpGs as compared to the HumanMethylation450 Bead Chip used in the above mentioned EWAS.

## Methods

### Samples

Seventy-six patients with OCD (37 female, age ± SD: 33.51 ± 10.92 years) of European descent (self-report up to third generation) were drawn from a larger sample of OCD patients (cf. [5, 9, 14]) recruited at the Psychosomatic Hospital Windach, Windach, Germany, between 2014 and 2017. OCD diagnosis was ascertained on the basis of a structured clinical interview according to DSM-IV criteria (SCID-I) by experienced psychiatrists and/or clinical psychologists. Severe somatic and neurological disorders, pregnancy, comorbid tic disorder, trichotillomania, skin-picking disorder or other current axis I diagnoses except for depression (*N* = 40), specific phobias (*N* = 5), social phobia (*N* = 3), panic disorder (*N* = 1), agoraphobia (*N* = 3) or post-traumatic stress disorder (*N* = 1) were excluded. Smoking status was ascertained in detail with the total number of cigarettes smoked per day (*N* = 14 smokers, mean no. of cigarettes/day ± SD: 10.5 ± 8.07). Fifty-eight patients (76.3%) received psychiatric medication at baseline (SSRIs: *N* = 44; SNRIs: *N* = 3; tricyclic antidepressants [TCA]: *N* = 9; mirtazapine: *N* = 4; bupropion: *N* = 2; lithium: *N* = 1; atypical neuroleptics: *N* = 14; pregabalin: *N* = 1; methylphenidate *N* = 1).

*N* = 76 healthy control probands were matched to the patient sample by age and sex (37 female, age ± SD: 33.25 ± 10.27 years). Controls were recruited within the framework of the Collaborative Research Centre TRR-58 “Fear, Anxiety, Anxiety Disorders” (cf. [15, 16]). Inclusion criteria were defined as European descent (self-report up to third generation), age at inclusion between 18 and 50 years, right-handedness, and fluency in German. Probands with a past or current DSM-IV axis I disorder as ascertained by experienced psychologists (Mini International Psychiatric Interview), past or current severe neurological or somatic disorders, current intake of centrally active medication, excessive alcohol (≥15 units/week), nicotine (≥20 cigarettes/day; *N* = 11 smokers, mean no. of cigarettes/day ± SD: 7.23 ± 6.88), and caffeine (≥4 cups/day) consumption, illegal drug use, or pregnancy were excluded.

This study was approved by the ethics committee of the University of Würzburg, Germany, and was conducted according to the ethical principles of the Helsinki Declaration. Written informed consent was obtained from all participants prior to participation.

### Blood sampling

EDTA blood was collected from all patients and controls. DNA was isolated using the FlexiGene DNA Kit (QIAGEN, Hilden, Germany) and stored at −80 °C until further processing.

### DNA methylation analysis

Aliquots of genomic DNA (250 ng) were treated with sodium bisulfite by means of the EZ-96 DNA Methylation Kit (ZymoResearch, Freiburg, Germany). The Infinium MethylationEPIC Kit was used to quantify DNA methylation at ~865,000 sites (Illumina, San Diego, USA). Hybridization and processing were performed according to the manufacturer’s instructions at Life & Brain, Bonn, Germany.

### Quality control and preprocessing of DNA methylation data

Processing and quality control of the raw methylation data was performed with a set of programs based on the CPACOR pipeline [17]. Principal component analysis of the control probes was calculated to be used for adjustment of technical measurement variance. Four samples were excluded due to outlying values for at least one of the control probe measures (mean ± 4*SD). The threshold for the sample call rate was set to 0.9 (0 samples). White blood cell type (WBC) sub-populations were estimated based on 100 CpG sites by the Houseman method [18] as implemented in the minfi R package [19]. CpGs potentially affected by cross-hybridization [20] were flagged. After data preprocessing and quality control, DNA methylation data from *N* = 68 OCD patients and *N* = 68 matched controls were available for analysis.

### Epigenome-wide association analyses

A two-step EWAS approach was performed as described previously [21]. In brief, DNA methylation *β*-values were regressed on the first three principal components of the control probes and the estimated WBC proportions for six cell types (CD8 T cells, CD4 T cells, natural killer cells, B-cells, monocytes, granulocytes). Then, a two-sided paired sample *t*-test for matched samples was performed. Statistical significance was defined as *p* < 5.77E-8, corresponding to a Bonferroni correction for the 865,859 evaluated CpG sites (0.05/865,859). Associations were reported as suggestive at *p* < 1E-5. Inflation was assessed by the genomic inflation factor lambda [22] and visual inspection of QQ-plots (see Supplementary Fig. S1). Significantly associated CpGs, termed quantitative trait methylation sites (QTMs), and suggestively significant CpG sites were checked for overlap with single nucleotide polymorphisms (SNP) (distance to SNP ≤ 5 base pairs (bp); European ancestry-based minor allele frequency ≥0.01) [23]. No such overlaps were found.

### In silico functional analyses

Data on blood–brain correlation of DNA methylation was looked up for all epigenome-wide significant QTMs in two databases: The BECon database [24] (https://redgar598.shinyapps.io/BECon/, consulted on 29 July 2021) and the IMAGE-CpG database [25] (https://han-lab.org/methylation/default/imageCpG, consulted on 29 July 2021). An additional online in silico analysis of the top ranked CpGs was performed using the Blood Brain DNA Methylation Comparison Tool based on the Illumina 450 K Beadchip array (available at https://epigenetics.essex.ac.uk/bloodbrain/; [26]; accessed 15 April 2022).

Targets of microRNAs identified to be differentially methylated were predicted using scanMiR [27]. Gene ontology enrichment analysis was performed using the TopGo algorithm (v.2.42.0) [28] and org.Hs.eg.db (3.12.0) with R. In brief, predicted targets of miR-12136 were filtered for those transcripts expressed as the highest 95% in induced cortical human neurons (dataset unpublished). Subsequently, the elim algorithm with Fisher’s exact test of TopGo was used to compare the top 100 expressed predicted target genes of miR-12136 against the genes belonging to the 95% highest expressed transcripts in human neurons (same dataset). Following the TopGo manual, multiple testing correction was not performed for the elim algorithm and mininum nodeSize set to 5.

## Results

### Case–control analysis and CpG localization

**Fig. 1:**
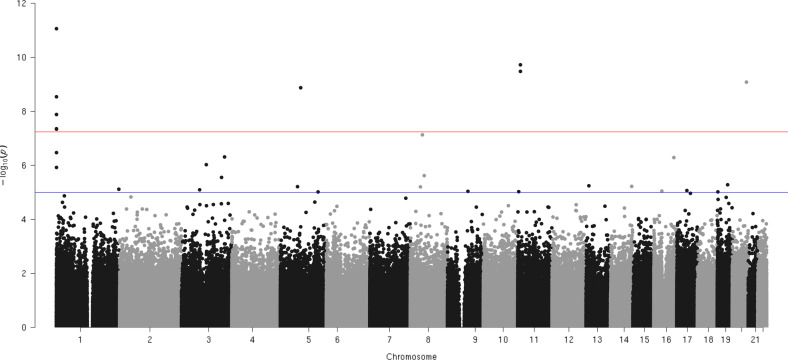
Manhattan plot of the matched case–control EWAS in OCD. Statistical significance (red horizontal line) was defined as *p* < 5.77E-8 (=0.05/865,859), corresponding to a Bonferroni correction for the 865,859 evaluated CpG sites. Dots for cg15174812 and cg11330075 are overlapping. Suggestive evidence was defined as *p* < 1E-5 (blue horizontal line).

**Fig. 2:**
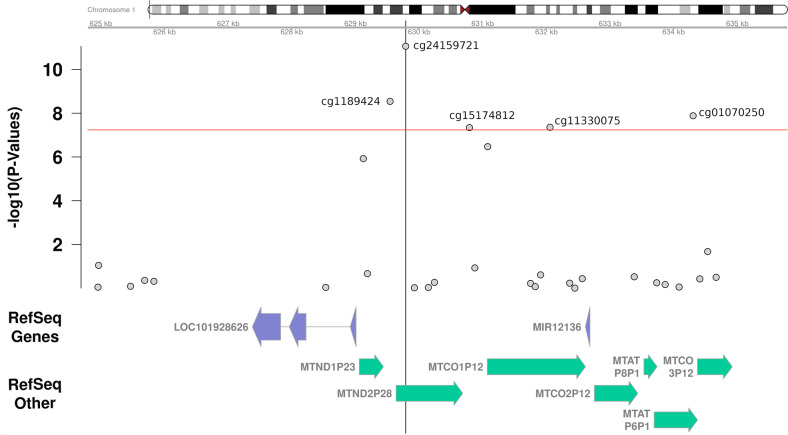
QTM locus on chromosome 1. Gene annotated display of all tested CpGs and their *p*-values in the region (± ~5 kb) of the top QTM (quantitative trait methylation) site (cg24159721) as a result of a case–control EWAS in OCD. RefSeqOther: not included in RefSeqCurated.

Epigenome-wide case–control association analyses resulted in nine epigenome-wide significant QTM sites (see Table 1 and Fig. 1). For all sites, DNA methylation was higher in the OCD group. In addition, suggestive *p*-values (*p* < 1E-05) were discerned for 21 further CpGs (see Supplementary Table S1). The CpG with the lowest *p*-value for the difference between the OCD and the control group was cg24159721 (*p* = 8.7E-12), located on chromosome 1: 629790 (Genome Reference Consortium Human Build 38). It maps 781 bp upstream of the start site of the noncoding RNA gene NR_125957.1 (RefSeq LOC101928626; Table 1 and Fig. 2). As an additional source of annotation, the presence of pseudogenes encoded in this region was evaluated given that the location of cg24159721 overlapped with a transcribed mRNA in human tissue studied as part of the GTEx Project [29]. Using an expanded, predicted subset of RefSeq genes as annotation, cg24159721 maps into the pseudogene MT-ND2 pseudogene 28 (*MTND2P28*) that according to the GTEx Project is transcribed ubiquitously, with high levels in all brain tissues studied (https://genome-euro.ucsc.edu/cgi-bin/hgc?hgsid=280873132_q4IEF2qZIsBsrjLuVjrrXNUyfywc&db=hg38&c=chr1&l=629639&r=630683&o=629639&t=630683&g=gtexGeneV8&i=MTND2P28).

**Table 1:** Epigenome-wide significant (*p* < 5.77E-8) QTM sites.

Rank *p*-value	CpG site	Chromosome: position^a^	Nearest RefSeq curated gene (relation)	Further RefSeq annotations	*p*-value	effect (Δ*β*)	Cohen’s *d*	Direction of differential methylation
1	cg24159721	chr1:629790	LOC101928626 (+781 bp of start site, upstream)	MT-ND2 pseudogene 28^b^	8.7E-12	0.032	1.00	Case > Control
2	cg05740793	chr11:10509544	MT-RNR2 like 8 (+358 bp of start site, upstream)	microRNA 4485	1.9E-10	0.028	0.91	Case > Control
3	cg20450977	chr11:10507916	MT-RNR2 like 8 (3’UTR)	microRNA 4485	3.3E-10	0.015	0.89	Case > Control
4	cg16267121	chr20:57360342	Ribonucleic Acid Export 1 (Body)	MT-RNR2 like 3	8.3E-10	0.017	0.87	Case > Control
5	cg15890734	chr5:80651415	Dihydrofolate Reductase (Body)	MT-RNR2 like 2	1.3E-09	0.015	0.85	Case > Control
6	cg11894324	chr1:629545	LOC101928626 (+536 bp of start site, upstream)	MT-ND2 pseudogene 28^b^	2.9E-09	0.023	0.83	Case > Control
7	cg01070250	chr1:634307	microRNA 12136 (+1894 bp of start site, upstream)	MT-CO1 pseudogene 12^b^	1.3E-08	0.026	0.79	Case > Control
8	cg11330075	chr1:632058	microRNA 12136 (−355 bp of start site, downstream)	MT-CO1 pseudogene 12^b^	4.4E-08	0.015	0.75	Case > Control
9	cg15174812	chr1:630792	microRNA 12136 (−1621 bp of start site, downstream)	MT-CO1 pseudogene 12^b^	4.6E-08	0.012	0.75	Case > Control

*QTM* quantitative trait methylation.

^a^Based on GRCh38.p13.

^b^RefSeqOther (not included in RefSeqCurated).

Only 245 bp away from cg24159721 and 536 bp upstream of the start site of *LOC101928626*, another QTM site was identified: cg11894324 on chr1:629545. This CpG also maps between the aforementioned *MTND2P28* and another pseudogene, MT-ND1 pseudogene 23 (*MTND1P23*). In the other direction, between *MTND2P28* and the MT-CO1 pseudogene 12 (*MTCO1P12*), maps the QTM cg15174812 (chr1:630792). This location is also 1.9 kb upstream of *LOC101928626* and 1.6 kb downstream of the microRNA 12136 gene (*MIR12136*). In addition, two further QTM sites (cg01070250, cg11330075) map into the same region flanking the *MIR12136* gene (see Fig. 2).

**Fig. 3:**
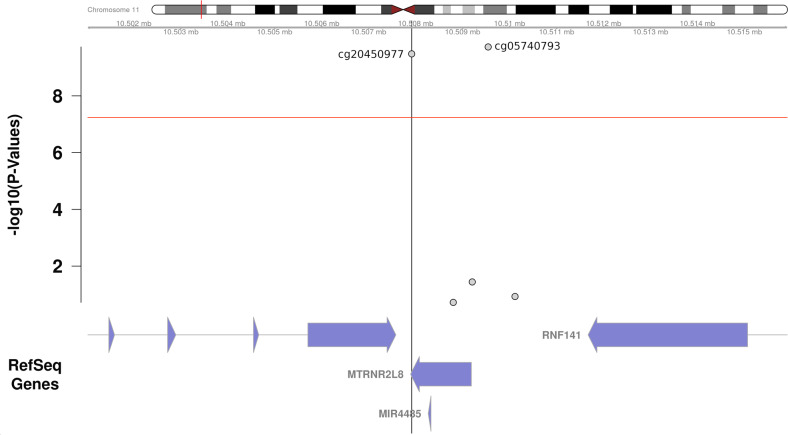
QTM locus on chromosome 11. Gene annotated display of all tested CpGs and their p-values in the region of the QTM (quantitative trait methylation) site cg20450977 (± ~7 kb) as a result of a case–control EWAS in OCD.

Two additional QTM sites were identified on chromosome 11: cg05740793 and cg20450977. These are located in the area of the protein coding MT-RNR2 like 8 gene, at coordinates 10509544 and 10507916, respectively. The former, cg05740793, maps 358 bp upstream of the start site of the gene, whereas the latter, cg20450977, maps to the end of the gene (3’UTR, see Fig. 3).

Another significant hit (cg16267121) was observed on chromosome 20 at position 57360342. It maps to an intron of the Ribonucleic Acid Export 1 (*RAE1*) gene. The nearest exon of the *RAE1* ends 1.8 kb upstream. The CpG is also located just 844 bp upstream of the start of the protein coding MT-RNR2 like 3 gene and is therefore positioned within the functionally relevant promoter region 1500 bp upstream of the transcription start site (TSS1500).

Lastly, the QTM cg15890734 maps to an intron of the Dihydrofolate Reductase gene at chr5:80651415. It is 1.9 kb downstream of the end of the nearest exon of this gene and 1.3 kb upstream of the processed pseudogene MT-RNR2 like 2.

### In silico functional analyses

The IMAGE-CpG provided correlation values for 8 of the 9 CpGs (cg20450977 missing). Three of the significant QTM sites showed small to moderate positive correlations between methylation in blood and brain: cg15890734 (rho = 0.31), cg11894324 (rho = 0.23), and cg05740793 (rho = 0.11). For two CpGs, cg15174812 and cg16267121, the correlation was almost zero (both rho=0.03). The remaining three CpGs had small to moderate negative brain-blood methylation correlations: cg11330075 (rho = −0.09), cg24159721 (rho = −0.27), cg01070250 (rho = −0.30). None of those values was significantly different from zero. The BECon data base did not include any of the significant QTM sites as these were conservatively excluded due to possible cross-reactivity.

An additional online in silico analysis of the top ranked CpGs using the Blood Brain DNA Methylation Comparison Tool revealed considerable blood–brain methylation correlations for CpGs cg15890734 and cg01070250 with respect to the available brain regions (prefrontal cortex [PFC], entorhinal cortex [EC], superior temporal gyrus [STG], and cerebellum [CER]), while for the remaining CpGs (if available on the Illumina 450 K array) no or only weak correlations were discerned: cg24159721 (blood-PFC: *r* = 0.00629, *p* = 0.958, blood-EC: *r* = 0.285, *p* = 0.0162, blood-STG: *r* = 0.191, *p* = 0.1, blood-CER: *r* = 0.353, *p* = 0.00255), cg05740793 (blood-PFC: *r* = −0.144, *p* = 0.222, blood-EC: *r* = −0.0102, *p* = 0.932, blood-STG: *r* = −0.183, *p* = 0.1, blood-CER: *r* = 0.169, *p* = 0.16), cg15890734 (blood-PFC: *r* = 0.841, *p* = 7.19e-21, blood-EC: *r* = 0.871, *p* = 5.78e-23, blood-STG: *r* = 0.847, *p* = 9.33e-22, blood-CER: *r* = 0.885, *p* = 1.33e-24), cg01070250 (blood-PFC: *r* = 0.257, *p* = 0.027, blood-EC: *r* = 0.407, *p* = 0.000427, blood-STG: *r* = 0.247, *p* = 0.0325, blood-CER: *r* = 0.51, *p* = 5.42e-06), cg15174812 (blood-PFC: *r* = −0.0555, *p* = 0.638, blood-EC: *r* = 0.0246, *p* = 0.839, blood-STG: *r* = 0.0144, *p* = 0.903, blood-CER: *r* = 0.0827, *p* = 0.493).

## Discussion

The present study applied a hypothesis-generating, epigenome-wide case–control approach in OCD using the Illumina Infinium MethylationEPIC BeadChip array. Nine epigenome-wide significant CpG sites were identified to be differentially methylated in blood between adult patients with OCD and healthy controls.

The top hit (cg24159721) as well as another CpG presently found to be differentially methylated in OCD (cg11894324) map to the upstream region of the noncoding RNA gene *LOC101928626* and the mitochondrially encoded NADH:ubiquinone oxidoreductase core subunit 2 (*MT-ND2*) pseudogene 28 (*MTND2P28*). While both genes are expressed in the brain, no study has associated them with mental disorders or OCD in particular so far. However, these two CpGs also map to the greater downstream region of the microRNA 12136 gene (*MIR12136*), and three further significant hits upstream (cg01070250) or downstream (cg11330075, cg15174812) of the *MIR12136* gene have been identified to be differentially methylated in OCD in the present study (cf. Fig. 2). Using scanMiR [27], the top 10 predicted targets of microRNA hsa-miR-12136 are: ZNF891, CREB1, FLRT2, RPS6KA5, MGAT4C, ZNF714, FGF13, FAM221A, SCAI and CLU4B. Most interestingly, the *FGF13* gene targeted by hsa-miR-12136 has been identified as one of the top hits in a previous epigenome-wide DNA methylation screen in OCD [13]. Genetic variation in the cAMP-response element binding protein (CREB1) has previously been reported to interact with G protein-activated K + channel 2 (GIRK2) gene variation in driving pronounced rumination and obsessional-compulsive personality disorder [30]. The Fibronectin Leucine-Rich Transmembrane protein 2 (*FLRT2*), expressed in the hippocampus, has been implicated in the development of synapse formation as well as learning and memory [31], and a rare copy number variant in the *MGAT4C* (Mannosyl (Alpha-1,3-)-Glycoprotein Beta-1,4-N-Acetylglucosaminyltransferase, Isozyme C) gene was found to be associated with neurocognitive impairment [32]. Potentially altered regulation of these genes driven by the presently observed differential methylation of *MIR12136* might thus affect neurocognitive domains that are central components of the neuropsychological profile of OCD [33–36]. An additional gene ontology (GO-Term) analysis of the top 100 predicted targets of miR-12136 (Supplementary Fig. S2 and Supplementary Table S2) revealed several terms associated with general microRNA processing as most significant. In order to mechanistically dissect this potential systemic impact of miR-12136 on general miRNA activity, future experiments could be conducted using for example miR-12136 manipulation (overexpression of mimics, inhibition with antisense oligonucleotides) in human-induced neurons followed by, e.g., smallRNA sequencing and morphological analyses of the neurons.

Two additional CpG sites presently found to be differentially methylated in OCD (cg05740793, cg20450977) are located in regions flanking the MT-RNR2 (humanin) like 8 gene (*MT-RNRL8*) (cf. Fig. 3), an isoform of the mitochondrial MT-RNR2 gene, which has been shown to function as a neuroprotective and antiapoptotic peptide [37, 38] and to be expressed in the brain [39]. A previous whole-transcriptome analysis in the brain identified an up-regulation of *MT-RNRL8* gene expression in both depression and depression-associated suicide in a presumably compensatory attempt to buffer stress [40]. Hypermethylation in the region of *MT-RNR2L8* as presently identified in OCD patients as compared to healthy controls—if exerting a downregulatory effect on transcription—might cautiously be interpreted as conferring insufficient neuroprotection and stress resilience in patients with OCD. Two further CpGs presently found to be differentially methylated in OCD as compared to controls map to the gene bodies of the ribonucleic acid export 1 (*RAE1*) gene (cg16267121) and the dihydrofolate reductase (*DHFR*) gene (cg15890734), respectively, which both have not been implicated in OCD pathogenesis so far. Interestingly, however, when using an alternate annotation method, these CpGs map directly to the regions of the MT-RNR2 (humanin) like 3 gene (*MT-RNRL3*) and MT-RNR2 (humanin) like 2 gene (*MT-RNRL2*), which are located in the intronic regions of *RAE1* (intron 4) and *DHFR* (intron 2), respectively [39]. So, in sum four CpGs presently identified to be differentially methylated in patients with OCD map to the humanin-like gene family suggesting further investigation of nuclear-encoded humanin isoforms with respect to OCD pathogenesis.

Despite several strengths such as high clinical and demographic homogeneity as well as strict inclusion and exclusion criteria minimizing the risk of confounding factors, the present study has some limitations. A general caveat while interpreting the present results arises from the fact that epigenome-wide DNA methylation was determined in blood, i.e., surrogate peripheral tissue, as no brain tissue can be ascertained in vivo in patients. Thus, no firm conclusions regarding central processes can be derived from the present results, particularly as an in silico search using the IMAGE-CpG database yielded only small, statistically not significant correlations if any. IMAGE-CpG, however, relies on unsorted brain tissue from patients undergoing neurosurgical resection for medically intractable epilepsy. An additional analysis using the Blood Brain DNA Methylation Comparison Tool [26] allowing for the interrogation of specific brain regions, i.e., the prefrontal cortex, the entorhinal cortex, the superior temporal gyrus and the cerebellum, revealed substantial blood–brain methylation correlations for CpGs cg15890734 and cg01070250. Still, none of the online available databases contains information on brain areas relevant for OCD such as the anterior cingulate gyrus, orbitofrontal cortex, ventral striatum, nucleus accumbens, caudate nucleus and putamen [41] and thus do not allow for a conclusive analysis. Given that no gene expression data was available, interpretation of the biological consequences of the observed differences in DNA methylation on gene transcription is limited. Also, while no statistical effect of psychiatric medication on DNA methylation was detected in the present sample, an influence of pharmacological treatment on the present results cannot be fully excluded given that 76% of patients received psychotropic medication. Furthermore, as epigenetic mechanisms have been suggested to mechanistically confer adaptation to life events at the interface between genetic and environmental influences [42–44], distant or recent adverse life events in the present sample could have introduced a bias. Also, comorbidity with other axis I diagnoses, particularly depression (*N* = 40), could in part have driven the present results, which therefore might not be specific for OCD. However, the present hits do not match significant loci identified in available epigenome-wide association studies in depression (e.g., refs. [45–52]). Finally, although on a similar scale to previous EWAS in mental disorders (e.g., refs. [12, 13, 21, 53–55]), the presently analyzed sample size of *N* = 68 per group was small, warranting independent replication in larger, better-powered samples.

In sum, the present EWAS revealed nine epigenome-wide significant QTMs in adult OCD. A particular role is suggested for differential methylation within the greater region of the microRNA hsa-miR-12136 (*MIR12136*) and humanin-like 2, 3, and 8 (*MT-RNRL2, MT-RNRL3*, *MT-RNRL8*) genes. Given the hypothesis-generating approach, the clinical translational value of the results is limited at present, however, they open up novel opportunities to interrogate the molecular biological pathogenesis of OCD. Although the presently observed effect sizes are statistically large (Cohen’s *d* = 0.75–1.00) relative to the variability within the sample, the range in methylation values is small (0.012–0.032, see Table 1). Thus, future studies are warranted to replicate the present findings in independent samples, to determine the functional relevance of the findings as well as their potential as peripheral surrogate markers of central nervous processes and to apply a longitudinal design in order to determine whether the present marks constitute state or trait markers of OCD. Peripheral epigenetic biomarkers potentially reflecting systemically measurable etiologically relevant processes might hold great potential for early identification of persons at risk for OCD and might potentially in the future inform both targeted preventive efforts as well as novel therapeutic approaches.

## Supplementary information

Electronic Supplementary Table S1

Legend to Electronic Supplementary Table S1

Electronic Supplementary Table S2

Legend to Electronic Supplementary Table S2

Electronic Supplementary Figure S1

Legend to Electronic Supplementary Figure S1

Electronic Supplementary Figure S2

Legend to Electronic Supplementary Figure S2
