# Supplementary material for: Epigenome-wide DNA methylation in obsessive-compulsive disorder
Source: Transl Psychiatry. 2022 Jun 1;12:221. doi: 10.1038/s41398-022-01996-w (PMC9160220; doi:10.1038/s41398-022-01996-w)
Supplement: Supplementary file 2 — Legend to Electronic Supplementary Table S1 [file 41398_2022_1996_MOESM2_ESM.docx]

# Electronic Supplementary Table S1: Annotated list of QTM sites and suggestive hits of the case-control EWAS in OCD.

Legend to Electronic Supplementary Table S1: The table includes the results in columns A-G, the coordinates for GRCh38.p13 – as reference to Table 1 and Figures 2 and 3 – in column H, an indicator for possible crossreactivity ^20^ in column I, and the information from the Illumina EPIC annotation file in columns J-AN, which is based on GRCh37.p13.
