# Supplementary material for: Epigenome-wide DNA methylation in obsessive-compulsive disorder
Source: Transl Psychiatry. 2022 Jun 1;12:221. doi: 10.1038/s41398-022-01996-w (PMC9160220; doi:10.1038/s41398-022-01996-w)
Supplement: Supplementary file 4 — Legend to Electronic Supplementary Table S2 [file 41398_2022_1996_MOESM4_ESM.docx]

# Electronic Supplementary Table S2: Gene ontology (GO-Term) analysis of the top 100 predicted targets of miR-12136
