# Supplementary figures and images for: Epigenome-wide DNA methylation in obsessive-compulsive disorder
Source: Transl Psychiatry. 2022 Jun 1;12:221. doi: 10.1038/s41398-022-01996-w (PMC9160220; doi:10.1038/s41398-022-01996-w)

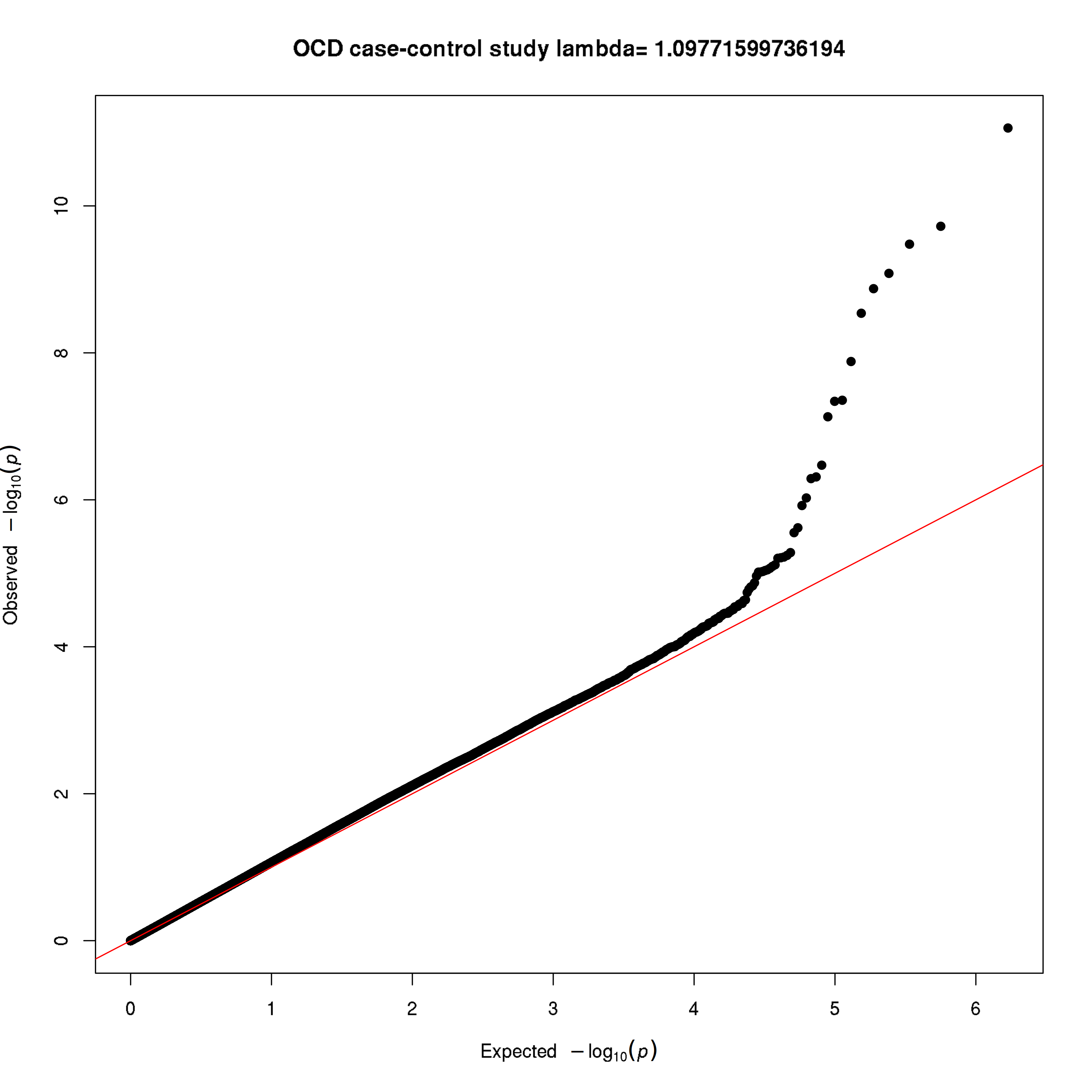

Supplement: Supplementary file 5 — Electronic Supplementary Figure S1 [file 41398_2022_1996_MOESM5_ESM.tif]

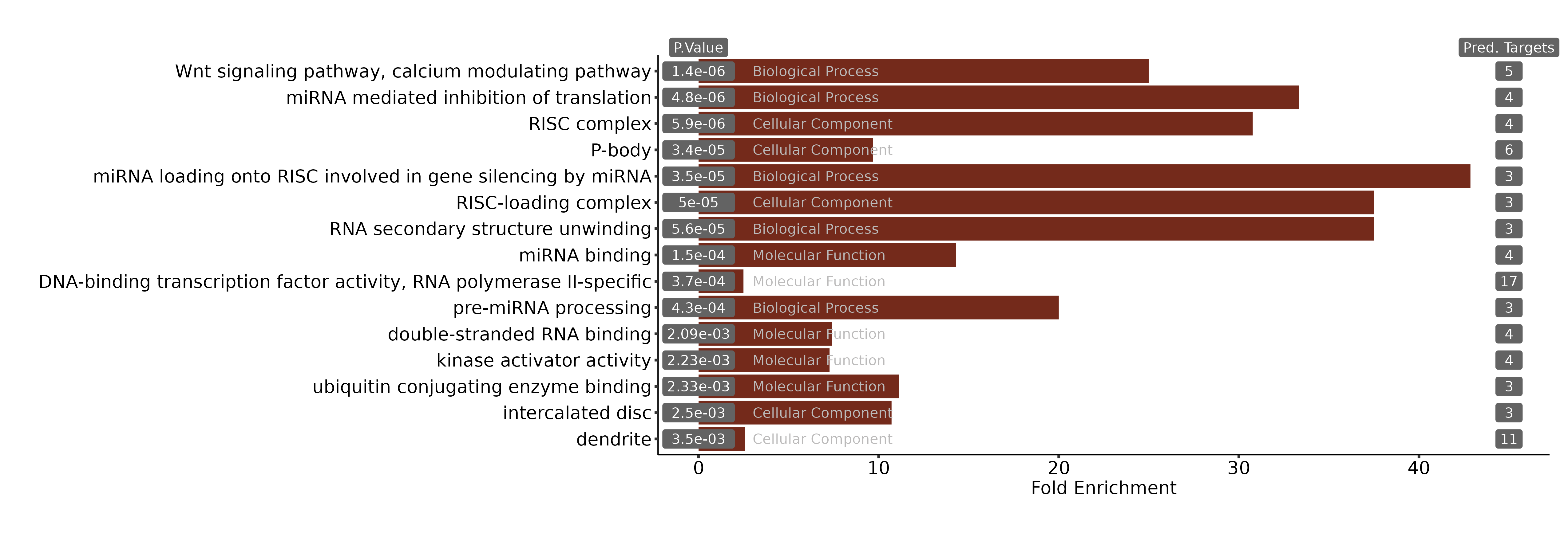

Supplement: Supplementary file 7 — Electronic Supplementary Figure S2 [file 41398_2022_1996_MOESM7_ESM.png]
