# Supplementary material for: Epigenome-wide DNA methylation in obsessive-compulsive disorder
Source: Transl Psychiatry. 2022 Jun 1;12:221. doi: 10.1038/s41398-022-01996-w (PMC9160220; doi:10.1038/s41398-022-01996-w)
Supplement: Supplementary file 6 — Legend to Electronic Supplementary Figure S1 [file 41398_2022_1996_MOESM6_ESM.docx]

**Electronic Supplementary Figure S1: QQ-Plot for the matched case-control EWAS in OCD**

Legend to Electronic Supplementary Figure S1: Quantile-Quantile (QQ) plot for the OCD case-control EWAS. QQ-plots visualize the deviation of the observed log10-transformed p-values from the theoretical null distribution (inflation). The inflation factor corresponding to the plot is lambda=1.098.
