# Supplementary material for: Epigenome-wide DNA methylation in obsessive-compulsive disorder
Source: Transl Psychiatry. 2022 Jun 1;12:221. doi: 10.1038/s41398-022-01996-w (PMC9160220; doi:10.1038/s41398-022-01996-w)
Supplement: Supplementary file 8 — Legend to Electronic Supplementary Figure S2 [file 41398_2022_1996_MOESM8_ESM.docx]

**Electronic Supplementary Figure S2: Gene Ontology (GO-Term) analysis of the top 100 predicted targets of miR-12136 against expressed genes in induced human cortical neurons**

Legend to Electronic Supplementary Figure S2: Depicted are the top 5 significant terms for each annotation (sorted by significance) with the number of predicted targets associated with these terms shown on the right side.
